# Endoscopic Bariatric Treatment with Duodenal-Jejunal Bypass Liner Improves Non-invasive Markers of Non-alcoholic Steatohepatitis

**DOI:** 10.1007/s11695-022-06150-5

**Published:** 2022-06-17

**Authors:** Thomas Karlas, David Petroff, Jürgen Feisthammel, Sebastian Beer, Matthias Blüher, Tatjana Schütz, Ralf Lichtinghagen, Albrecht Hoffmeister, Johannes Wiegand

**Affiliations:** 1 Department of Medicine II, Division of Gastroenterology, Leipzig University Medical Center, Liebigstraße 20, 04103 Leipzig, Germany; 2 Clinical Trial Centre Leipzig, University of Leipzig, Härtelstraße 16/18, 04107 Leipzig, Germany; 3 Integrated Research and Treatment Center (IFB) AdiposityDiseases, University of Leipzig, Philipp-Rosenthal-Str. 27, 04103 Leipzig, Germany; 4 Helmholtz Zentrum München, Helmholtz Institute for Metabolic, Obesity and Vascular Research (HI-MAG), University of Leipzig and University Hospital Leipzig, Leipzig, Germany; 5 Institute of Clinical Chemistry, Hannover Medical School, Carl-Neuberg-Str.1, 30625 Hannover, Germany; 6 Department of Medicine II, Division of Hepatology, Leipzig University Medical Center, Liebigstraße 20, 04103 Leipzig, Germany

**Keywords:** Duodenal-jejunal bypass liner, Endobarrier, Fibroscan, Liver elastography, NASH, Diabetes

## Abstract

**Purpose:**

People with obesity often develop non-alcoholic fatty liver disease (NAFLD) and are at high risk of progression to non-alcoholic steatohepatitis (NASH). Few therapies are effective other than bariatric surgery. We therefore analyzed data from duodenal-jejunal bypass liner (DJBL) patients regarding steatosis, fibrosis, and NASH.

**Methods:**

Consecutive DJBL patients with type 2 diabetes underwent standardized assessments up to device removal at 48 weeks. These included aspartate and alanine transaminase (AST, ALT), controlled attenuation parameter (CAP, for steatosis), and liver stiffness measurement (LSM, for fibrosis). The NAFLD fibrosis score (NFS), fibrosis-4 score (FIB4), and enhanced liver fibrosis (ELF) test were also used to assess fibrosis and the Fibroscan-AST (FAST) score to assess NASH. Mixed models were used and missing data were accounted for with multiple imputation.

**Results:**

Thirty-two patients (18 female, mean age 55.1, mean BMI 40.2 kg/m^2^) were included. After 48 weeks, the change compared to baseline with 95% CI was a factor 0.74 (0.65 to 0.84) for AST, 0.63 (0.53 to 0.75) for ALT, and a difference of − 0.21 (− 0.28 to − 0.13) for FAST, all with *p* < 0.001. Fibrosis based on LSM, NFS, and ELF did not change whereas FIB4 exhibited slight improvement. Eight DJBL were explanted early due to device-related complications and eight complications led to hospitalization.

**Conclusions:**

One year of DJBL therapy is associated with relevant improvements in non-invasive markers of steatosis and NASH, but not fibrosis, and is accompanied by a substantial number of complications. Given the lack of alternatives, DJBL deserves further attention.

**Graphical abstract:**

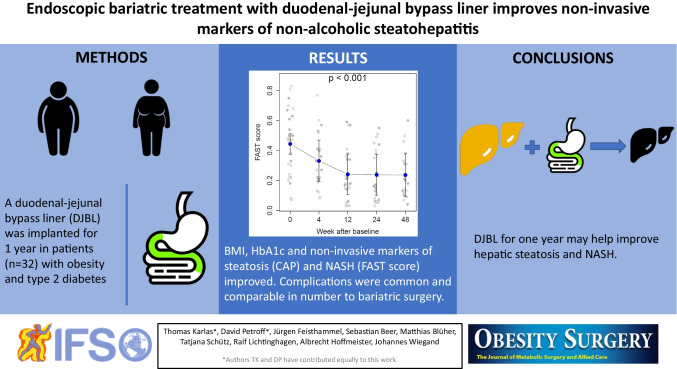

## Introduction

Obesity has become a worldwide concern and is often associated with non-alcoholic fatty liver disease (NAFLD) and particularly in combination with the metabolic syndrome and/or type 2 diabetes mellitus (T2DM) may progress to non-alcoholic steatohepatitis (NASH). For NASH alone, lifestyle intervention is commonly the first approach, though with limited success [1, 2]. Selected patients may benefit from pharmacotherapy [3], but guidelines have yet to recommend such a therapy. A proven concept for the many patients when the aforementioned options fail is bariatric surgery, which has recently been shown to improve both NASH and overall survival [4, 5]. Bariatric surgery, however, is associated with potentially severe harms requiring lifelong surveillance [6, 7] and is not opted for by many patients although reversal is possible [8]. Reversible therapeutic approaches concentrate on excluding duodenal-jejunal resorption [9]. The duodenal-jejunal bypass liner (DJBL) is an impermeable flexible tube-implanted endoscopically, anchored in the duodenal bulb and extending for 60 cm, thereby preventing all contact between chyme and mucosa in this area of the jejunum. The DJBL has been shown to reduce BMI and improve glycemic control over a twelve month treatment period [10–12]. Small sample sizes and ethical concerns with serial liver biopsies mean that few data are available for assessing the effect of DJBL on NAFLD. Non-invasive surrogates for hepatic fibrosis and steatosis have been implemented in a single-case series in DJBL patients [13], but impact on NASH has not been studied. Recent advances combining liver stiffness measurement (LSM), steatosis estimation with controlled attenuation parameter (CAP), and aspartate aminotransferase (AST) now provide a first non-invasive marker—the FAST score [14]. We thus collected data on consecutive patients undergoing DJBL to study weight change and glycemic markers. These can be expected to affect NASH activity and fibrosis.

## Methods

### Ethic Approval and Informed Consent

All patients gave written informed consent for prospective collection of their clinical data, which was approved by the Ethics Committee at the University of Leipzig (reg. no. 352/08-B-ff and no. 006/09 ff).

### Study Cohort

From September 2013 to September 2016, DJBL treatment was available at our institution for patients who fulfilled at least one of the following conditions: (a) BMI ≥ 30 kg/m^2^ and T2DM, (b) BMI ≥ 30 kg/m^2^ and at least one comorbidity, (c) BMI ≥ 35 kg/m^2^. Consecutive patients underwent a structured pre-treatment assessment and were monitored thoroughly using a standardized protocol until device explantation, planned for 12 months.

### Duodenal-Jejunal Bypass Liner Procedure and Follow-up

The DJBL (GI Dynamics, USA) is an impermeable tube of 60 cm length. It was placed endoscopically in the upper gastrointestinal tract and fixed in the duodenal bulb using a nitinol anchor under general anesthesia and X-ray control following the manufacturer’s recommendations [15]. The standard treatment period was 48 weeks with regular follow-up visits at baseline, 4, 12, 24, and 48 weeks.

Data collection included LSM, anthropometry with calculation of the body mass index (BMI), and routine laboratory testing including liver function tests and glycated hemoglobin (HbA1c). A cut-off of HbA1c > 7.5%, which is the upper limit of the target range recommended by the German Diabetes Association, indicated insufficient diabetes control [16]. Serum samples were immediately stored at − 20° C for analysis of enhanced liver function (ELF) score.

### Liver Stiffness Measurement and Non-invasive Assessment of Hepatic Steatosis

LSM was performed at each visit with a Fibroscan 502 equipped with both M- and XL-probes. Measurements were performed according to the manufacturer’s recommendations using the Boursier criteria for definition of reliable measurements [17]. At the LSM measuring site, skin-to-liver capsule distance (SCD) was determined with a linear ultrasound transducer. SCD ≥ 25 mm indicated the use of the XL probe [18]. XL probe was also used in case of invalid M probe measurements. During follow-up, the probe of the baseline examination was used for all examinations.

Controlled attenuation parameter (CAP) was used for non-invasive estimation of hepatic steatosis as described before [19]. Because the CAP algorithm was not yet available for the XL probe at the beginning of the study, CAP XL values were calculated by the manufacturer Echosens from stored raw data in these cases.

Cut-off values of LSM > 8.2 kPa and CAP > 331 dB/m were chosen to define the risk of advanced fibrosis and steatosis, respectively [20].

### Indices of Hepatic Fibrosis and Inflammatory Activity

ELF score is a marker of hepatic fibrosis. Analyses were performed from stored serum samples (baseline, 12, 24, and 48 weeks) according to the manufacturer’s instructions in February 2017 as described before [21]: Serum concentrations of tissue inhibitor of metallo-proteinases-1 (TIMP-1), amino-terminal propeptide of type III procollagen (PIIINP), and hyaluronic acid (HA) were used to calculate the ELF score. ELF values of > 7.7 and > 9.8 defined intermediate and high fibrosis risk [22].

From routine laboratory data, we calculated the NAFLD fibrosis score (NFS) and the FIB4-score (FIB4) as described in Blank et al. [23]. For FIB4, values below 1.3 for patients under the age of 60 and 2.0 for those above that age indicated low risk of advanced fibrosis and values above 3.25 for all ages indicated high risk [24]. For NFS, values below − 1.455 for patients between the ages of 35 and 60 and 0.12 for those above that age indicated low risk of advanced fibrosis and values above 0.675 for all ages indicated high risk [25].

The Fibroscan-aspartate-aminotransferase score (FAST) is a novel marker to predict the risk of active NASH (NASH activity index ≥ 4) with significant fibrosis (≥ F2). The score was calculated retrospectively according to the published algorithm from Newsome et al. [14]. A low (0.35; sensitivity 90%) and a high (0.67; specificity 90%) cut-off were chosen to assess low, intermediate, and high risk of fibrotic NASH.

### Statistical Analysis

All analyses were carried out and graphics rendered with the software R, version 4.0.4. Mixed models with restricted maximum likelihood were used to evaluate repeated measures in which time was treated as a categorical variable. Confidence intervals from the mixed models at a given point in time were found with a profiling method and contrasts between adjacent points in time were determined using the “multcomp” package with the Westfall method for *p*-value adjustment [26]. Categorical data were compared to baseline using an exact Wilcoxon signed-rank test, with the Pratt treatment of zeros as provided by the “coin” package [27]. To compare if treatment in an abstract sense or changes in BMI were more strongly associated with changes in clinical parameters, mixed models with maximum likelihood and time as a continuous parameter or changes in BMI were evaluated and then formally compared with the likelihood ratio test from Vuong and where we provide values for Akaike’s information criterion (AIC) [28]. In each of the models, the value of BMI and the dependent variable at baseline were taken as covariates. Missing data for scores were accounted for with multiple imputation using 50 sets using the software package “mice” [29]. Missing data for categories were imputed with last observation carried forward, where the known drawbacks are attenuated in the case of ordinal data. As a sensitivity analysis, the most pessimistic possible scenario (“pessimum”) was considered, in which all missing data were replaced by the most pathological category. *p*-values below 0.05 were considered significant.

## Results

**Fig. 1:**
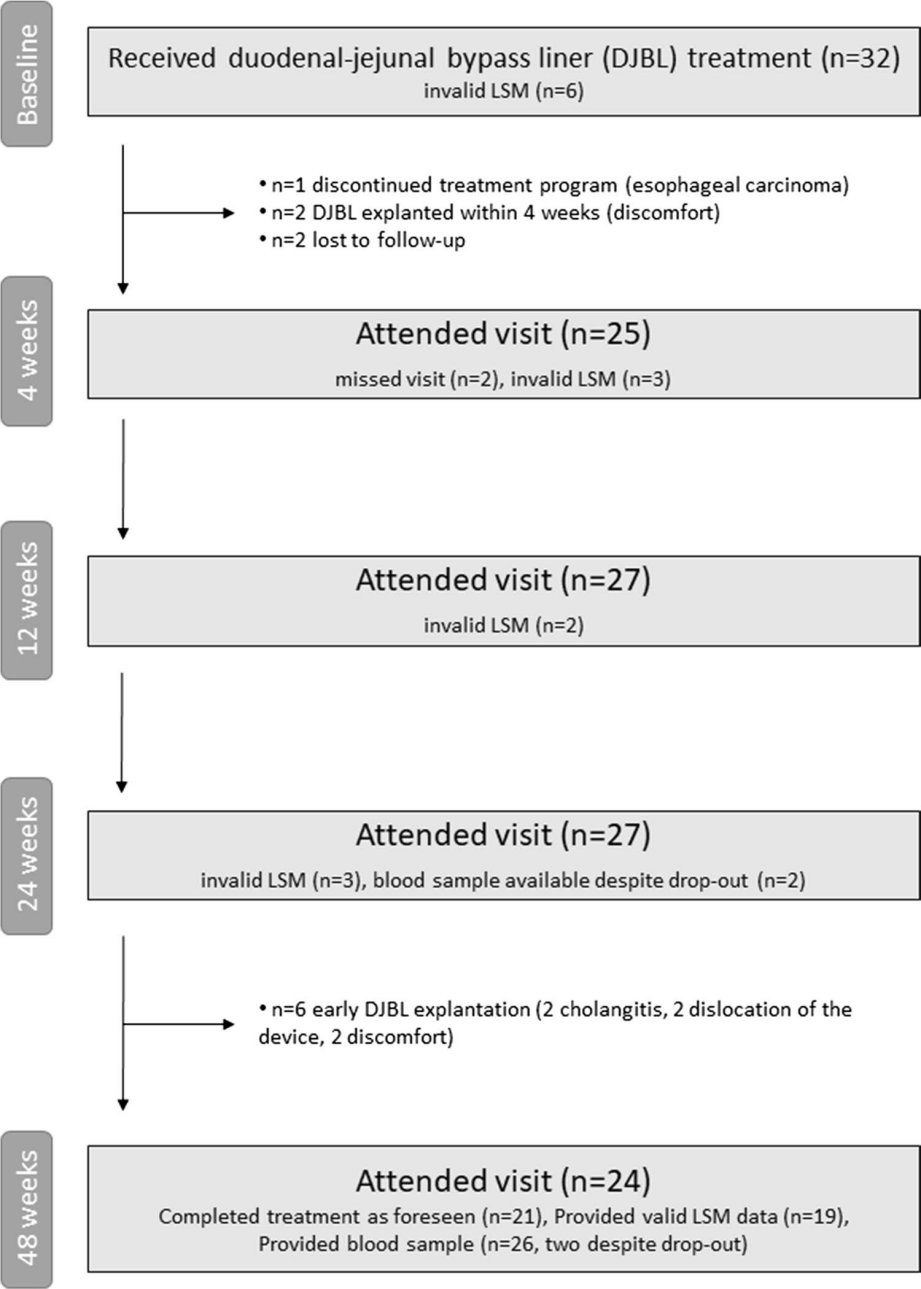
Patient flow and availability of data

Thirty-two patients were registered in the database. Figure 1 shows the patient flow. All patients had T2DM despite the fact that this was not required according to the inclusion criteria and further characteristics at baseline are presented in Table 1. At baseline, 26 valid vibration-controlled transient elastography (VCTE) measurements were available, 25 with the XL probe and one with the M probe. Invalid LSM was associated with high BMI.

**Table 1:** Patient characteristics at baseline. Entries are mean ± standard deviation, median (interquartile range), or numbers (percentages)

Number of patients	32 (100%)
Sex (female)	18 (56%)
Age (years)	55.1 ± 6.6
Height (m)	1.69 ± 0.09
Weight (kg)	116 ± 34
BMI (kg/m^2^)	40.2 ± 10.0
< 30	2 (6%)
≥ 30 and < 35	7 (22%)
≥ 35 and < 40	13 (41%)
≥ 40 and < 50	7 (22%)
≥ 50 and < 60	1 (3%)
> 60	2 (6%)
HbA1c (%)	7.3 ± 1.2
> 7.5	13 (41%)
LSM (kPa)*	6.2 [5.5, 6.6]
≥ 8.0	3 (12%)
CAP (dB/m)*	357 ± 40
≥ 331	19 (73%)
AST (in ULN)	0.75 [0.64, 1.11]
> 1	10 (31%)
ALT (in ULN)	0.90 [0.72, 1.22]
> 1	14 (44%)
Liver scores	
FAST*	0.45 ± 0.22
Low NASH risk	10 (38%)
Intermediate NASH risk	11 (42%)
High NASH risk	5 (19%)
FIB-4	1.42 ± 0.67
Low risk of advanced fibrosis	16 (50%)
Further investigation	15 (47%)
High risk of advanced fibrosis	1 (3%)
NAFLD fibrosis score	0.01 ± 1.32
Advanced fibrosis excluded	5 (16%)
Further investigation	19 (59%)
Advanced fibrosis likely	7 (22%)
Alternative fibrosis assessment needed	1 (3%)
ELF**	9.08 ± 0.76
Low risk of fibrosis	0 (0%)
Intermediate risk of fibrosis	24 (86%)
High risk of fibrosis	4 (14%)

^*^Data available for 26 patients

^**^Data available for 28 patients

**Fig. 2:**
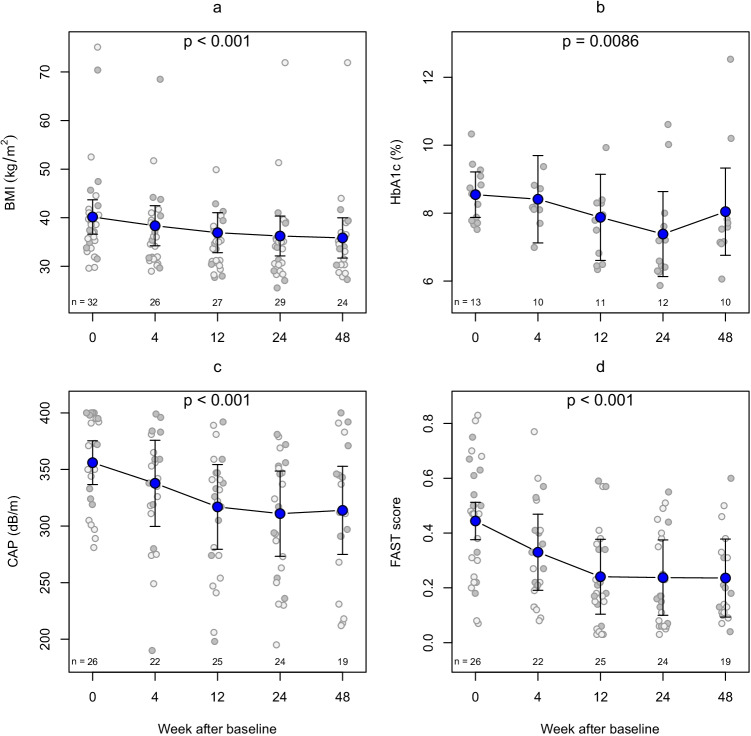
The course of various parameters is shown from DJBL implantation (week 0) to explantation (week 48). The gray points show raw data with the lighter shade representing patients with HbA1c < 7.5% at baseline. Note that panel b only presents data from those with HbA1c ≥ 7.5% at baseline. The blue dots are estimates from the linear mixed model and the whiskers represent 95% confidence intervals

Figures 2A and B show that the metabolic parameters BMI and HbA1c depend on time, as expected, and where that change is significant using a global test. Considering only the contrast from baseline to 48 weeks, the reduction in BMI was − 4.3 kg/m^2^ (95% CI − 4.9 to − 3.7) corresponding to a change in weight by 10.8% (95% CI 9.2 to 12.3), and for HbA1c, it was − 0.5% (95% − 1.1 to 0.1). There was a significant reduction in AST and ALT (both *p* < 0.001) by a factor 0.74 (95% CI 0.65 to 0.84) and 0.63 (95% CI 0.53 to 0.75), respectively. Figures 2C and D also show that these changes are accompanied by significant and relevant reductions in steatosis by − 42 dB/m (95% CI − 62 to − 22) and in NASH risk on an absolute scale by − 0.21 (95% CI − 0.28 to − 0.13). No difference between adjacent points in time is significant. The FAST score can be interpreted as a probability for NASH and a mixed model after multiple imputation shows that the odds ratio (relative scale) at week 24 for NASH compared to baseline is 0.28 (95% CI 0.17 to 0.47, *p* < 0.001) and at week 48 is 0.32 (95% CI 0.18 to 0.56).

**Fig. 3:**
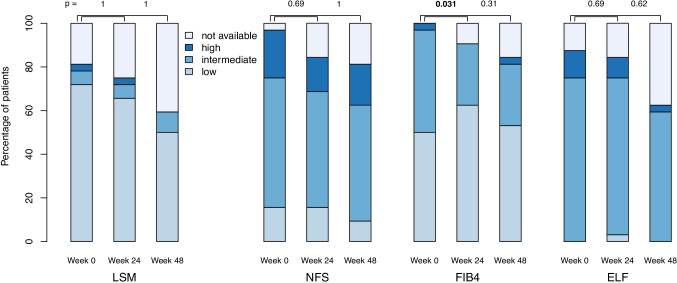
Risk of fibrosis categories based on liver stiffness measurement (LSM) with three different scores (NFS non-alcoholic fatty liver disease fibrosis score, FIB4 fibrosis 4 score, ELF enhanced liver fibrosis test) are shown as functions of time.

VCTE-based (LSM) and laboratory (NFS, FIB4, ELF-score) markers of fibrosis risk are presented in Fig. 3. Baseline fibrosis risk depends largely on which score is chosen, but within each score, there is little evidence for change over time with the exception of FIB4.

Although the LSM-based categories did not exhibit change over time, the LSM value itself did to a small extent. Compared to baseline, the value, which was analyzed on a logarithmic scale, was lower at week 24 by a factor of 0.83 (95% CI 0.69 to 0.99, *p* = 0.041) and at week 48 by 0.86 (95% CI 0.70 to 1.05, *p* = 0.14). The NFS score changed by − 0.20 (95% CI − 0.66 to 0.26, *p* = 0.39) points at week 24 and 0.06 (95% CI − 0.42 to 0.53, *p* = 0.81) points at week 48, where the minus sign indicates a clinical improvement. The FIB4 score, also analyzed on a logarithmic scale, decreased by factor 0.82 (95% CI 0.70 to 0.95, *p* = 0.010) at week 24 and 0.86 (95% CI 0.74 to 1.01, *p* = 0.059) at week 48. The ELF score changed by − 0.11 (95% CI − 0.36 to 0.14, *p* = 0.40) points at week 24 and − 0.02 (95% CI − 0.30 to 0.25, *p* = 0.88) points at week 48, where the minus sign again indicates clinical improvement.

### Association Between Clinical Parameters and BMI

There is no significant evidence suggesting that changes in BMI are more strongly associated with changes in HbA1c or LSM than time on therapy (AIC_time_ = 378, AIC_BMI_ = 363, *p* = 0.12 for HbA1c, and AIC_time_ =  − 180, AIC_BMI_ =  − 185, *p* = 0.15 for LSM). On the other hand, this is the case for changes in CAP (AIC_time_ = 1471, AIC_BMI_ = 1446, *p* = 0.0029). According to the mixed model, for every kg/m^2^ decrease in BMI, CAP decreases by 9.9 dB/m (95% CI 7.0 to 12.7 dB/m).

For the liver fibrosis scores, there is also no significant evidence suggesting stronger association with changes in BMI than with time on intervention (AIC_time_ = 325, AIC_BMI_ = 328, *p* = 0.71 for NFS, AIC_time_ = 181, AIC_BMI_ = 176, *p* = 0.28 for FIB4). The ELF score could not be analyzed in such a model because of the small number of points in time and the fairly discrete nature of the underlying data. For the FAST score, there is slight but not significant evidence of stronger association with changes in BMI: AIC_time_ =  − 144, AIC_BMI_ =  − 166, *p* = 0.087. According to the mixed model, for every kg/m^2^ decrease in BMI, FAST decreases by 0.041 (95% CI 0.030 to 0.052).

### Correlation Between Changes in BMI, HbA1c, and FAST

Although the largest reductions in BMI, HbA1c, and FAST were considerable (9.3 kg/m^2^, 3.6% and 0.62 points), correlations among the three were quite weak: *r* = 0.27 (95% CI − 0.11 to 0.58, *p* = 0.16) for BMI vs. HbA1c, *r* = 0.16 (95% CI − 0.28 to 0.55, *p* = 0.47) for BMI vs. FAST, *r* = 0.46 (95% CI 0.04 to 0.74, *p* = 0.032) for HbA1c vs. FAST. Restricting attention to patients with baseline HbA1c > 7.5%, the above estimate become *r* = 0.24 (95% CI − 0.39 to 0.72, *p* = 0.45) for BMI vs. HbA1c, *r* = 0.54 (95% CI − 0.19 to 0.89, *p* = 0.13) for BMI vs. FAST, and *r* = 0.38 (95% CI − 0.38 to 0.83, *p* = 0.32) for HbA1c vs. FAST.

### Complications

Three patients ended the DJBL therapy within 4 weeks, two as a result of discomfort and the third after being diagnosed with esophageal cancer (Fig. 1). Six further early removals occurred during the second half of therapy (2 dislocations, 2 instances of gastrointestinal discomfort or pain, and 2 cases of cholangitis). Overall, eight patients were hospitalized as a result of complications and ten had gastrointestinal pain, nausea, or vomiting. There were 18 patients without complications.

## Discussion

Our study provides first longitudinal data on steatosis, NASH, and liver fibrosis modification for the 1 year duration of an endoscopic duodenal-jejunal bypass procedure. There is evidence for a decline in steatosis and NASH risk over time, but little evidence for changes in fibrosis. A substantial number of patients had complications, many of them leading to early explantation of the DJBL.

NASH has not yet been studied systematically in DJBL patients. A small DJBL pilot study with serial measurements in 13 patients did observe significant modulation of fibrosis and steatosis surrogates [13]. These results are difficult to generalize, however, given the unusually high prevalence of advanced liver disease. Our study now provides first data from a larger and typical cohort of T2DM patients at risk for NASH.

There exists an accepted rationale for comparing this DJBL therapy with gastric bypass surgery because both eliminate resorption in the upper small intestine and reduce passage time to the ileum. This affects glucose homeostasis through complex hormonal changes, e.g., in glucagon-like peptide-1 (GLP-1) [30, 31]. These effects may be comparable to changes induced by medical therapy, i.e., GLP-1 receptor agonists such as semaglutide 0.4 mg subcutaneously daily, a promising candidate for NASH therapy [3]: At one year, our data showed a reduction in CAP, AST, ALT, and LSM by − 42 dB/m, and factors of 0.74, 0.63, and 0.83, respectively. These are fairly similar to the reductions seen at 72 weeks in a recent double-blind phase 2 trial in patients with biopsy-confirmed NASH for the highest daily dose of 0.4 mg semaglutide used there, namely − 39 dB/m, 0.52, 0.42, and 0.72, respectively [3]. In bariatric surgery, studies with a varying proportion of gastric bypass found that CAP was reduced by 62 to 75 dB/m and mean or median LSM by a factor of 0.7 to 0.8 [32–35]. However, a direct comparison of bariatric surgery, DJBL, and GLP-1 agonists has not yet been conducted and merits a prospective trial with special attention to modulation of glucose homeostasis.

The complications we saw both shortly after DJBL implantation and especially after 6 months are comparable in magnitude to those reported in bariatric surgery and other duodenal bypass liner cohorts. For example, one randomized controlled trial with 50 gastric bypass patients found that 22% had required hospitalization by 1 year [36]. A meta-analysis on duodenal bypass jejunal liners found 10 studies that reported adverse events and that 15.7% of the patients had severe adverse events (SAE), though the length of follow-up varied widely among the studies [37]. To compare with patients from the aforementioned semaglutide trial, SAE were reported in 15%, though only 5% on 0.4 mg daily had severe gastrointestinal events [3]. The relatively high rate of complications reflects the invasiveness of the DJBL endoscopic procedure including the risks of general anesthesia and the considerable incidence of associated infectious sequelae, e.g., cholangitis. Note also that many complications occurred after 6 months and previous studies have commonly chosen to deploy the liner for less than 6 months (see, e.g., Ruban et al. [38] including Table 2 as well as the discussion therein) so that the risk–benefit ratio may be more favorable if explanted earlier. Therefore, DJBL therapy with the current device requires careful balancing of risks and possible benefits for the individual patient [39]. It may especially be worth considering in patients with indication for bariatric surgery, who are adverse to an irreversible alteration of bowel anatomy.

Fibrosis progression or regression in NAFLD is a slow process. In our study, the fibrosis specific ELF score—in the limited number of available data sets—did not change over a period of 48 weeks. In the semaglutide trial, reductions of − 0.56 points were observed after 72 weeks though no change in fibrosis categories was seen [3]. In our data, the NFS score also showed no change, whereas LSM and FIB4 did exhibit slight reductions. LSM not only reflects tissue matrix properties (fibrosis), but also more transitory states of vascular and tissue pressure (features of inflammation) [40], making it difficult to ascribe these changes to fibrosis alone. FIB4 depends largely on AST, which may explain the observed changes. These problems with the fibrosis surrogates may also account for the large discrepancies seen at baseline in the risk categories assigned.

One limitation of our study is the fairly low case number, which is typical for observational studies focussing on bariatric treatment [37]. Also typical for such studies is a non-negligible amount of missing data that are highly informative, e.g., after early explantation or invalid LSM, but also much less informative, e.g., if loss is due to a missed study visit. We treated missing data with mixed models and imputation, but especially for the patients with early explantation, the result may be overly optimistic estimates. The population includes a wide spectrum of metabolic morbidity and the modest mean change in BMI means that extrapolation to more extreme situations such as following surgical gastric bypass require caution. As a retrospective analysis of prospectively collected data, a further limitation is that we did not measure parameters of glucose homeostasis modification and that diabetes therapy has been changing since the start of our study. However, the retrospective analysis permitted us to implement the FAST score in the longitudinal setting. Finally, liver biopsy as the gold standard for fibrosis staging is clearly not suited to monitor progression, especially in patients with severe obesity.

In conclusion, we have presented important pilot data that demonstrate the potential benefit of duodenal-jejunal bypass for NASH improvement based on a non-invasive marker. Future studies with larger case numbers should verify NASH improvement and explore potential additive effects of duodenal-jejunal bypassing and GLP-1 agonists.
